# Safety assessment of slope on in-service dump under severe dry–wet cycles at high-altitude

**DOI:** 10.1038/s41598-023-44002-z

**Published:** 2023-10-09

**Authors:** Jianjun Dong, Hao Jiang, Di Yang, Ke Gao

**Affiliations:** 1 College of Safety Science and Engineering, Liaoning Technical University, Huludao, 125100 Liaoning China; 2 Key Laboratory of Mine Thermodynamic disasters and Control of Ministry of Education, Liaoning Technical University, Huludao, 125100 Liaoning China

**Keywords:** Environmental sciences, Natural hazards, Engineering

## Abstract

This study aimed to evaluate the safety of dump slopes in high-altitude areas subjected to severe dry–wet cycles. The slope of No. 1 and No. 2 in-service dumps in limestone mining areas for cement in high-altitude mining areas is taken as the research object. The unsaturated soil shear strength and matrix suction distribution equations were imported based on the unsaturated–saturated seepage theory. Therefore, the evolution characteristics of the unsaturated–saturated seepage field in the dump are analyzed by numerical calculation, and the safety state of the dump slope is evaluated. The results indicated the following rules: under the action of four dry–wet cycles, the surface soil of the dump slope changes from an unsaturated state to a saturated state. Furthermore, with the increase in the times of the dry–wet cycle, the maximum vertical displacement of the No. 1 and No. 2 dump slopes increased. The numerical calculations of the maximum cumulative vertical displacement of the slope were consistent with the actual monitoring data. The factor of safety of the dump slope decreased continuously with the increase in the times of dry–wet cycles. Nevertheless, it still met the safety and stability standards. It was concluded that the slope of the in-service dump remains stable after enduring four severe cycles of dry–wet.

## Introduction

The Qinghai-Tibet Plateau exhibits a distinctive plateau monsoon that arises from the interaction of summer heat low-pressure and winter cold high-pressure systems. Controlled by westerlies during the winter half-year, the region experiences arid conditions, whereas the influence of the southwest and southeast monsoon belts during the summer half-year results in wet conditions, with precipitation primarily concentrated in this period. At the onset of the wet season, high-altitude soil undergoes a transition from a dry to a humid state. However, due to the altitude, the solar radiant heat is stronger than that at low-altitude, and the evaporation after rain remains high even in the wet season. Following a prolonged dry season, heavy rainfall can cause severe alternation of dryness and wetness. Additionally, the frequent and intense alternation of dryness and wetness from continuous rainfall and high evaporation during the wet season exacerbates this phenomenon further. Hence, significant and severe alternation of dryness and wetness is a typical feature of high-altitude soil on the Qinghai-Tibet Plateau.

The alternation of unsaturated soil between residual water content and saturated water content represents a process of severe dryness and wetness alternation. Conversely, during the continuous rainfall dry–wet cycle with bidirectional alternations of dryness and wetness, unsaturated soil experiences a recurring process of low to medium moisture uptake and desorption cycles at medium to low hydraulic gradients as it transforms from residual to saturated water content. High-altitude engineering projects present a challenge due to severe dry–wet alternations that lead to a reduction in soil shear strength and increase the risk of landslides^1,2^. The potential consequences of such disasters on important national engineering projects such as the Sichuan-Tibet Railway (Linzhi-Lhasa section) and engineering construction (Ya'an-Linzhi section) cannot be ignored. Therefore, conducting research on the safety status evaluation of dump slopes in high-altitude areas under severe dry–wet cycle conditions is of great significance for ensuring the economic security and development of high-altitude regions.

In recent years, landslides caused by rainfall have occurred frequently^3–5^. Numerous scholars have investigated the safety and stability of slopes under rainfall conditions. Generally, the continual drop of soil shear strength significantly contributes to slopes' failure during rainfall^6–8^. For example, Nguyen. et al.^9^ studied the influence of spatial variability of soil properties such as shear strength parameters and hydraulic parameters on slope stability during rainfall infiltration, and found that the spatial variability of shear strength parameters influences slope stability most. Li et al.^10^ discussed the mechanisms through which excavation and rainfall affect slope stability and established a numerical model to simulate rainfall infiltration. They found rainfall plays a dominant role in triggering landslides, as water infiltrates into newly formed cracks caused by excavation, thereby reducing the shear strength of the slope. Liao^11^ determined that the shear strength and factor of safety of high-expansive rock slopes decrease with an increasing number of dry–wet cycles, and the shear strength stabilizes after eight dry–wet cycles. Cai et al.^12^ established a layered slope infiltration model and used numerical simulations to demonstrate that the relationship between rainfall intensity and permeability coefficient significantly affects the distribution of soil volumetric water content. Kristo et al.^13^ , with the FOS of slope as the starting point, discovered that the increase in rainfall intensity due to climate change significantly reduces the FOS of slope and negatively impacts slope stability. Yang et al.^14^ investigated the shear strength characteristics of expansive soil and found that the cohesion of the expansive soil slope decreases exponentially with an increase in dry–wet cycles, leading to a decline in soil shear strength and slope collapse. An increase in overlying pressure can effectively inhibit the strength decay. Yang et al.^15^ obtained the crack evolution law of expansive soil slopes under the effect of dry–wet alternation through indoor experiments. With an increase in dry–wet alternation times, the soil cohesion and matrix tension decrease, and the soil structure is damaged, generating cracks that provide infiltration channels for water and thereby affect the stability of the expansive soil slope. Lee et al.^16^ examined the failure mechanism associated with rainfall-triggered landslides utilizing flume experiments. Their findings establish that the gradual increase in rainfall infiltration results in a proportional rise in the volumetric water content of the soil, consequently leading to the gradual attenuation of the matric suction. This attenuation, in turn, induces a decline in the soil's overall strength, ultimately culminating in the instability of the slope soil.

**Figure 1:**
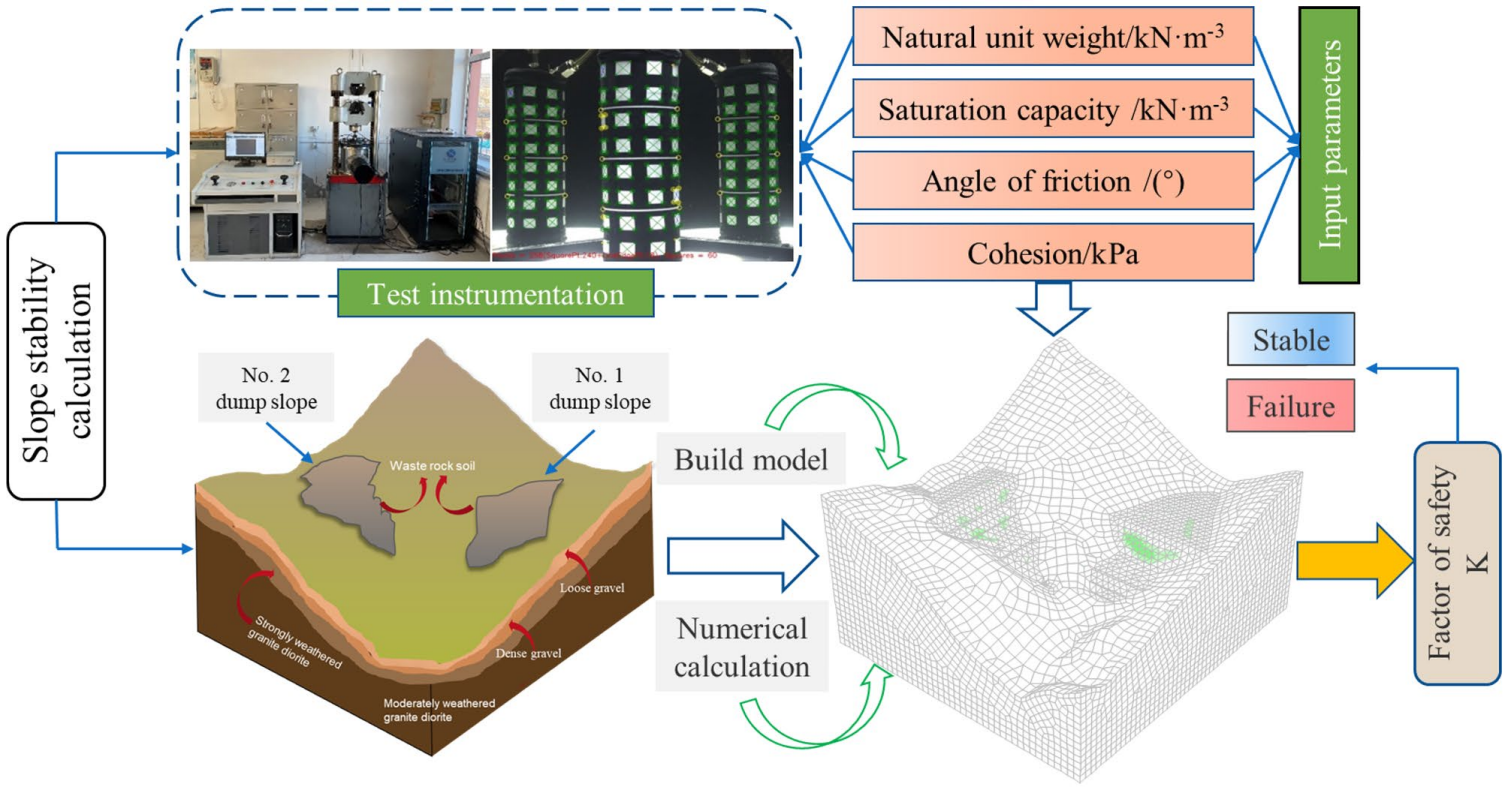
The evaluation model of dump slope stability state using numerical method.

However, the studies mentioned above primarily focus on the influence of conventional rainfall infiltration on the stability of non-high-altitude slopes and do not consider the intense dry–wet cycle effect caused by the special monsoon climate in high-altitude areas. Therefore, the author researches the in-service soil disposal site slopes No. 1 and No. 2 affiliated with the limestone mine in the Ma'amu mining area of Tibet. Based on experimental data and rainfall data from 2021, the non-saturated soil shear strength formula is used to establish a numerical model of the soil disposal site slope for analysis. This study evaluates the safety status of the in-service dump slopes in the high-altitude mining area under an intense dry–wet cycle (Fig. 1), providing valuable insights into the disaster prevention and control of high-altitude dump slopes.

## Principles of numerical calculation of dry–wet cycles

Based on previous research results, the basic principles of the theory, equations, and methods used in the numerical simulation of the safety and stability of the dump slope are as follows^17^:

### Unsaturated soil shear strength theory

The shear strength of the slope soil determines the stability of the slope. The following formula is used for the unsaturated soil shear strength:

1 $$\tau_{f} = [c^{\prime} + (\sigma_{n} - u_{a} )\tan \phi^{\prime}] + S_{e} (u_{a} - u_{{\text{w}}} )\tan \phi^{\prime} \,$$

where $\tau_{f}$ is the shear stress on the failure surface of the soil (kPa), $c^{\prime}$ is the effective cohesion (kPa), $\sigma_{n}$ is the normal stress (kPa), $u_{a}$ is the pore air pressure (kPa), $\varphi^{\prime}$ is the effective internal friction angle (°), $S_{e}$ is the effective saturation, $u_{{\text{w}}}$ is the pore water pressure (kPa).

When the pore water pressure and pore air pressure of the soil are equal, Eq. (1) represents the formula for saturated shear strength.

The total cohesion expression is as follows:

2 $$c_{t} = c^{\prime} + S_{e} \left( {u_{a} - u_{{\text{w}}} } \right)\tan \phi ^{\prime}$$

where $c_{t}$ represents the total cohesion (kPa), not the total stress cohesion.

### Unsaturated soil seepage theory

The unsaturated–saturated soil seepage equation is as follows:

3 $${\varvec{q}}_{i} = - k_{r} (S)K_{ij} h_{{,}_{j}} = k_{r} (S)K_{ij} {\mathbf{[}}\psi + \psi_{z} {\mathbf{]}}_{,j}$$

where ${\varvec{q}}_{i}$ is the unit flow vector, $k_{r} (S)$ is the relative permeability coefficient, with 0 < $k_{r} (S)$ < 1 for unsaturated soil and $k_{r} (S)$ = 1 for saturated soil, $K_{ij}$ is the permeability coefficient tensor, $h_{{,}_{j}}$ is the hydraulic gradient, $\psi$ is the pressure head, $\psi { = }u_{{\text{w}}} {/}\gamma_{{\text{w}}}$ (kPa), $\gamma_{{\text{w}}}$ is the unit weight of water (kN/m^3^) and $\psi_{z}$ is the position head (kPa).

The equation for unsaturated soil permeability coefficient is as follows:

4 $$k_{u} = kS_{e}^{0.5} [1 - (1 - S_{e}^{1/m} )^{m} ]^{2}$$

where $k_{u}$ is the unsaturated hydraulic conductivity (m/s), $k$ is the saturated permeability coefficient (m/s), $m$ is the fitting parameter.

### Matric suction distribution law

Under steady-state conditions, the matric suction in the vertical direction of unsaturated flow in natural unsaturated soil layers follows Darcy's law, and the specific discharge equation is as follows:

5 $$q = - k\exp [ - \beta \psi_{h} \gamma_{w} ][d\psi_{h} /dz + 1]$$

where $\beta$ is the variation rate of soil permeability coefficient dependent on matric suction(kPa^−1^), $\psi_{h}$ is the matric head, $\psi_{h} = (u_{a} - u_{{\text{w}}} )/\gamma_{{\text{w}}}$.

Integrating Eq. (5) with the boundary condition is *z* = 0, the suction force can be obtained as:

6 $$u_{a} - u_{{\text{w}}} = ( - 1/\beta )\ln [(1 + q/k)\exp ( - \beta z\gamma_{w} ) - q/k]$$

When the system is under static pressure conditions and *q* = 0, the matric suction shows a linear distribution:

7 $$u_{a} - u_{{\text{w}}} { \,=\, }z\gamma_{{\text{w}}}$$

### Strength reduction method

The strength reduction method is applied to calculate the FOS of slope of a soil heap. The reduction of soil shear strength is equivalent to the reduction of soil cohesion and internal friction angle, and the expressions are as follows:

8 $$c_{F} = c_{t} /F_{r} = [c^{\prime} + s_{e} (u_{a} - u_{{\text{w}}} )\tan \varphi^{\prime}]/F_{r}$$

9 $$\varphi_{F} = \tan^{ - 1} \left( {\tan \varphi^{\prime}/F_{r} } \right)$$

where $c_{F}$ is the reduced cohesion (kPa), $\varphi_{F}$ is the reduced internal friction angle (°), and $F_{r}$ is the reduction coefficient.

The expression for the FOS is as follows:

10 $$K{ = }\int\limits_{{0}}^{{1}} {\left( {c^{\prime} + \left[ {\sigma_{n} - u_{a} + s_{e} (u_{a} - u_{{\text{w}}} )\tan \varphi^{\prime}} \right]} \right)} dl/\int\limits_{0}^{1} {\tau dl}$$

### Mohr–Coulomb elastic–plastic stiffness matrix

The elastic–plastic stiffness matrix of the numerical calculation model for the dump slope under unsaturated–saturated state changes is derived from the Mohr–Coulomb elastic–plastic model. In classical elastic–plastic theory, strain $\varepsilon$ is divided into elastic strain $\varepsilon^{e}$ and plastic strain $\varepsilon^{p}$. The elastic part is calculated according to Hooke's law, and the plastic part is calculated according to plastic theory, using the incremental method:

11 $$d\varepsilon = d\varepsilon^{e} + d\varepsilon^{p}$$

In classical elastic–plastic theory, the expressions for stress increment and strain increment are:

12 $$d\sigma = \left[ D \right]_{ep} d\varepsilon = \left( {\left[ D \right]_{e} - \left[ D \right]_{p} } \right)d\varepsilon$$

where $d\sigma$ is the stress increment, $d\varepsilon$ is the strain increment, $\left[ D \right]_{ep}$ is the elastic–plastic stiffness matrix, $\left[ D \right]_{e}$ is the elastic matrix and $\left[ D \right]_{p}$ is the plastic stiffness matrix.

By deduction and formal manipulation, Eq. (12) is transformed into Eq. (13):

13 $$d\sigma = \left[ D \right]_{e} d\varepsilon { - }\frac{{\left[ D \right]_{e} \frac{\partial g}{{\partial \sigma }}\left( {\frac{\partial f}{{\partial \sigma }}} \right)^{T} \left[ D \right]_{e} \left( {d\varepsilon } \right)}}{{ - \frac{\partial f}{{\partial H}}\left( {\frac{\partial H}{{\partial \varepsilon^{p} }}} \right)^{T} \frac{\partial g}{{\partial \sigma }} + \left( {\frac{\partial f}{{\partial \sigma }}} \right)^{T} \left[ D \right]_{e} \frac{\partial g}{{\partial \sigma }}}}$$

where $g$ is the plastic potential function, $f$ is the yield function, and $H$ is the hardening parameter, $H = H\left( {\varepsilon^{P} } \right)$.

By combining Eqs. (13) and (14), the final expression for the elastic–plastic stiffness matrix $\left[ D \right]_{ep}$ is obtained.

14 $$\left[ D \right]_{ep} = \left[ D \right]_{e} - \frac{{\left[ D \right]_{e} \frac{\partial g}{{\partial \sigma }}\left( {\frac{\partial f}{{\partial \sigma }}} \right)^{T} \left[ D \right]_{e} }}{{\frac{\partial f}{{\partial H}}\left( {\frac{\partial H}{{\partial \varepsilon^{p} }}} \right)^{T} \frac{\partial g}{{\partial \sigma }} + \left( {\frac{\partial f}{{\partial \sigma }}} \right)^{T} \left[ D \right]_{e} \frac{\partial g}{{\partial \sigma }}}}$$

In the equations

$$\begin{aligned} \frac{\partial f}{{\partial \sigma }} & = \frac{\partial g}{{\partial \sigma }} = \frac{\partial f}{{\partial \sigma_{m} }}\frac{{\partial \sigma_{m} }}{\partial \sigma } + \frac{\partial f}{{\partial \sqrt {J_{{_{2} }} } }}\frac{{\partial \sqrt {J_{{_{2} }} } }}{\partial \sigma } + \frac{\partial f}{{\partial J_{{3}} }}\frac{{\partial J_{3} }}{\partial \sigma }{ = }\frac{{\sin \varphi^{\prime}}}{3}\frac{{\partial \sigma_{m} }}{\partial \sigma } + \cos \theta_{\sigma } \times \left[ {1 + \tan \theta_{\sigma } + \frac{1}{\sqrt 3 }\sin \varphi^{\prime}\left( {\tan 3\theta_{\sigma } - \tan \theta_{\sigma } } \right)} \right]\frac{{\partial \sqrt {J_{{_{2} }} } }}{\partial \sigma } \\ & \quad + \frac{{\sqrt 3 \sin \varphi^{\prime} + \sin \varphi^{\prime}\cos \theta_{\sigma } }}{{2J_{{2}} \cos 3\theta_{\sigma } }}\frac{{\partial J_{3} }}{\partial \sigma }{\text{ + s}}_{e} \sin \varphi^{\prime}\frac{\partial s}{{\partial \sigma }}, \\ \end{aligned}$$

$\frac{\partial f}{{\partial H}}\left( {\frac{\partial H}{{\partial \varepsilon^{p} }}} \right)^{T} \frac{\partial g}{{\partial \sigma }}{ = 0}$, $J_{2}$, $J_{3}$ are the second and third invariants of the deviatoric stress tensor, respectively, $\theta_{\sigma }$ is the Lode angle, expressed in degrees.

## Numerical calculation scheme for dry–wet cycles

### Project overview

The cement-grade limestone mine dump site of Mamu Mining Area is located in Sangri County, Tibet Autonomous Region, with an average altitude of up to 4000 m, belonging to a typical high-altitude region. The project site is located on the slope accumulation landform at the foot of the mountain, with numerous ridges and valleys characterized by steep ridges and narrow valleys. The southern dump site includes No. 1 dump and No. 2 dump, separated by only one ridge and less than 200 m apart. According to the topographical conditions, the dump sites are stacked in a step-by-step manner from low to high. No. 1 dump currently has a total dump height of about 47 m, divided into two steps, with a slope angle of about 34 degrees and step heights of 9 m and 38.3 m, respectively. The platform width is 243.3 m and about 740,000 cubic meters of waste rock and soil have been dumped. No. 2 dump has been closed, and the overall terrain is relatively flat with an elevation of about 4000 to 4004 m, lower than the design elevation of 4011.1 m, with a capacity of 5.975 million cubic meters. Currently, it has a dump height of about 132 m, divided into seven steps with step heights ranging from 10 to 50 m, a platform width of about 10 m, and slope angles of the steps ranging from 32 to 40 degrees. The overall dump slope is about 23 degrees, which is a low-risk area for debris flow and belongs to a complex site. Based on the above conditions, it is determined that the southern dump site should be classified as a second-class dump site^18^.

**Figure 2:**
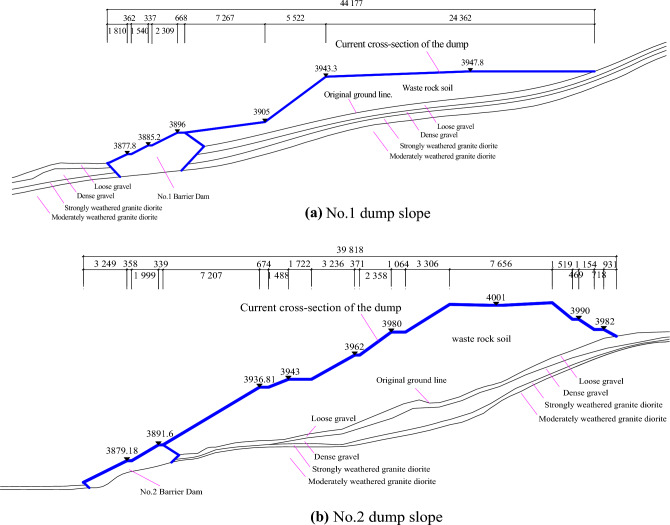
Profile of dump slope in-service.

As per the survey data, the soil dumping area exhibits a vertical distribution of surface to subsurface layers, comprising three primary layers (Fig. 2). The first layer, Q_4_^ml^ or artificial fill layer, is primarily constituted by powdery clay and discarded limestone blocks. It serves as the principal component of the dumping slope. The second layer, Q_4_^al+pl^ or Quaternary alluvial layer, is composed of crushed stone soil, categorized into two layers of loosely and slightly densely packed crushed stones, depending on the degree of compaction. The content of slightly dense crushed stones ranges from 55–65%, and their particle sizes are predominantly in the range of 2–15 cm, with a small proportion larger than 15 cm. This layer consists of granite, feldspar, and sandstone, among others, distributed throughout the surface of the entire mountainous region. The third layer, γδK_1_ or Early Cretaceous, represents the entire dumping area and is mainly composed of granite rock, classified into strongly weathered and moderately weathered granite based on their degree of weathering.

### Numerical modeling

**Figure 3:**
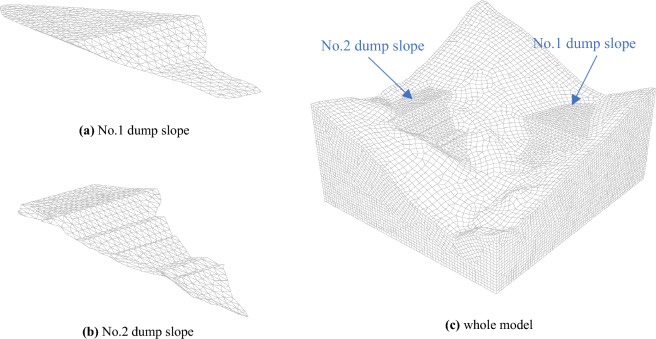
The geometric model.

A numerical simulation geometric model (Fig. 3) was constructed to simulate the in-service dump slope excavation volume. The whole model has dimensions of 665.2 m × 755.4 m × 483.3 m, and the mountain body is modeled using a hexahedral mesh while the dump slope is modeled using a tetrahedral mesh. The model is divided into 149,488 elements and 562,099 nodes. Two boundary conditions are applied: (1) a seepage boundary with the upper surface of the model set as permeable and the surrounding area set as impermeable^19,20^, and (2) a mechanical boundary with the upper surface set as free and the bottom and four side surfaces set as normal fixed constraints. To accurately simulate the mechanical characteristics of the rock-soil body and the applicable range of the constitutive model, the Mohr–Coulomb elastic–plastic model is selected, which is compatible with the mechanical characteristics of the rock-soil body and suitable for simulating and analyzing the stability of the slope. Additionally, an isotropic seepage model is chosen for the seepage analysis.

**Figure 4:**
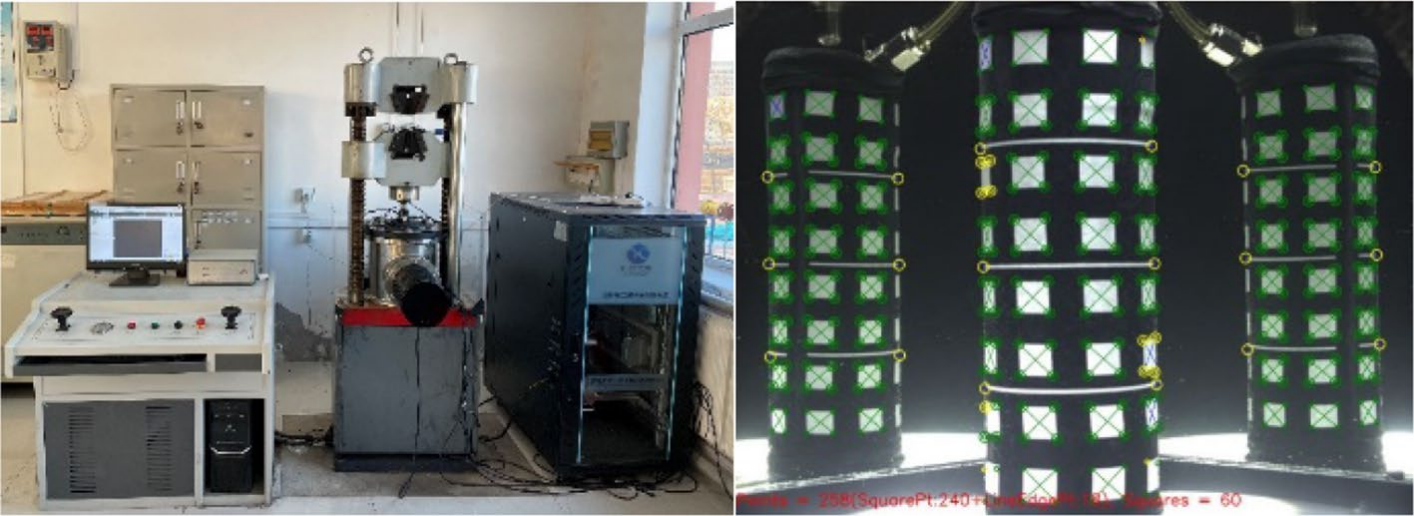
Test instrumentation.

Physical and mechanical properties of the soil body of the dump slope are obtained by conducting triaxial shear tests and Soil–Water Characteristic Curve (SWCC) tests^21^. The parameters for shear strength and SWCC model derived from the test results are utilized as numerical calculation model parameters. Test results, including shear strength parameters and SWCC model parameters, are presented in Tables 1 and 2, respectively. The testing equipment employed is depicted in Fig. 4.

**Table 1:** Basic strength parameters of dump slope.

Engineering material	Natural unit weight (kN m^−3^)	Saturation capacity (kN m^−3^)	Angle of friction (°)	Cohesion (kPa)
Barrier dam	20.5	–	35	5
Waste rock soil	21.5	22.5	25.3	4
Loose gravel	20.5	21.0	20	8
Dense gravel	21.0	22.0	22	10
Strongly weathered granite diorite	22.5	22.8	30	40
Moderately weathered granite diorite	25.2	25.5	38	500

**Table 2:** Basic parameters of SWCC model.

(kPa^−1^)	$k_{s}$ (m s^−1^)	$\gamma_{w}$ (kN m^−3^)	$\theta_{s}$ (%)	$\theta_{r}$ (%)	$\alpha$	*m*	*n*
0.005	2.5 × 10^–6^	9.8	50.29	11.90	0.06	0.15	4

### Selection of seepage parameters

**Figure 5:**
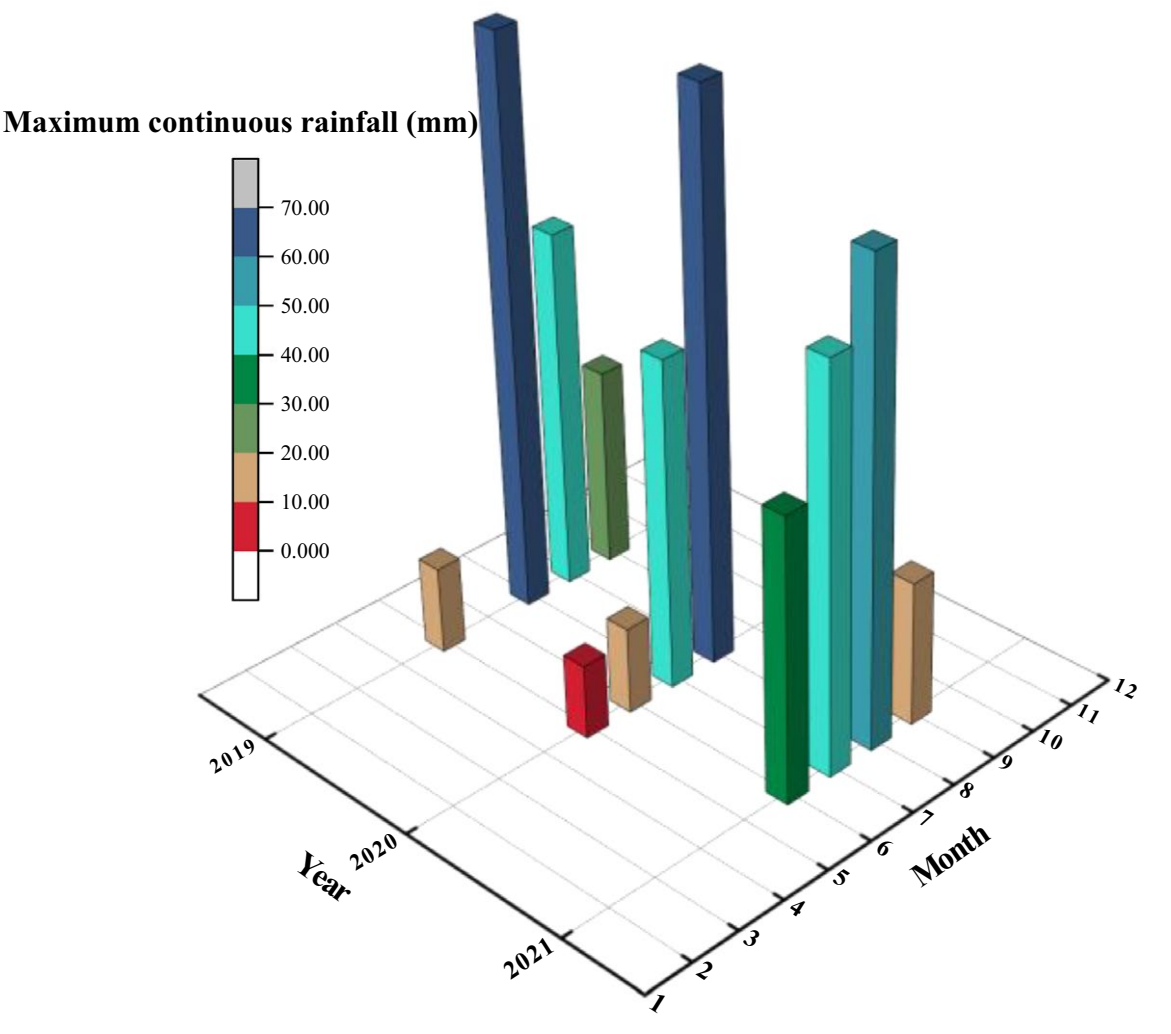
The maximum continuous rainfall occurring in 2019–2021.

Based on the analysis of meteorological data collected from Sangri County, Shannan City, Tibet, China, spanning from 2019 to 2021, it was discovered that the region experienced its highest maximum consecutive rainfall (lasting for at least 2 days) mainly between June and September, with the most substantial rainfall recorded in 2021 (Fig. 5). The findings from this study will inform the selection of appropriate seepage parameters for the numerical model.

**Figure 6:**
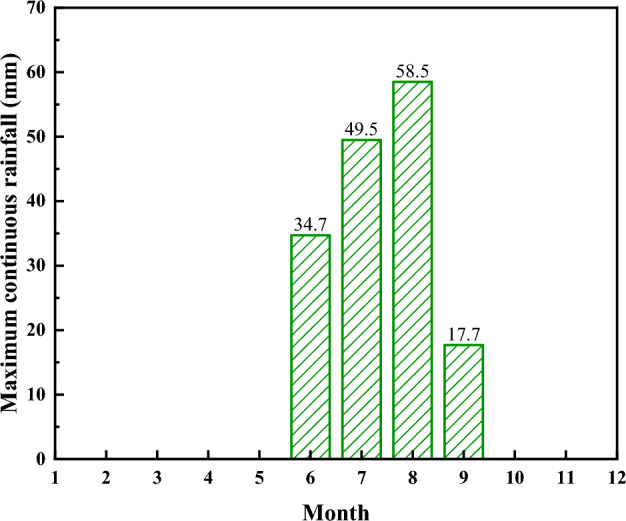
The maximum continuous rainfall occurring in 2021.

During the period from June to September 2021, there was a sustained precipitation event (Fig. 6), which occurred on specific dates ranging from June 6 to June 8, 2021, July 3 to July 6, 2021, August 21 to August 25, 2021, and September 6 to September 7, 2021. The maximum durations of consecutive rainfall for the four months were 34.7 mm, 49.5 mm, 58.5 mm, and 17.7 mm, respectively. Therefore, the dry–wet cycle times were set to four. In consideration of the most hazardous situation, where a single rainfall event occurs every three hours, the intensity of the dry–wet cycles (*Q*) was determined as 3.2 × 10^−6^ m/s, 4.6 × 10^−6^ m/s, 5.4 × 10^−6^ m/s, and 1.6 × 10^−6^ m/s, respectively.

**Figure 7:**
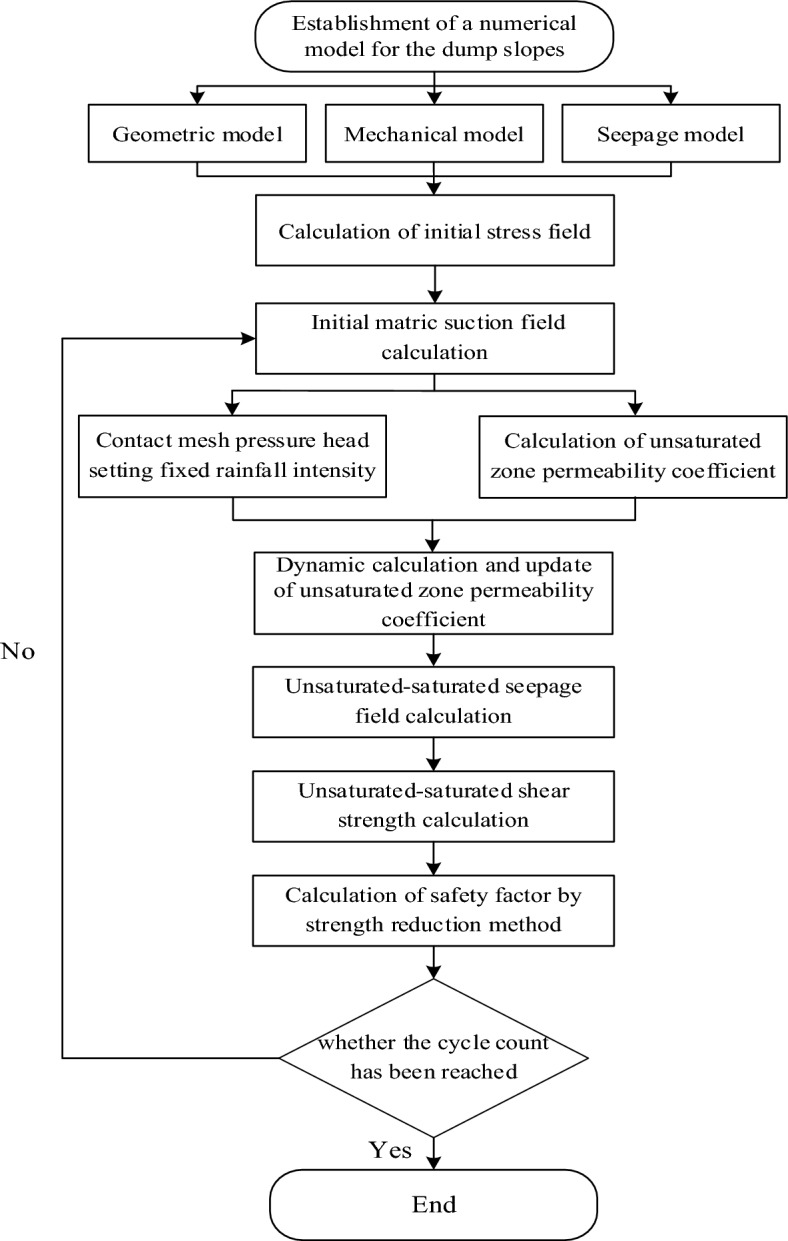
The flowchart of the dry–wet cycles calculation process.

To simulate the calculation of a severe dry–wet cycle, it was necessary to use Fish language for secondary development. The focus of secondary development is to achieve the following calculations in the model: (1) Unsaturated–saturated seepage calculation. (2) Dynamic update of permeability coefficient in unsaturated zone. (3) Unsaturated–saturated shear resistance Intensity calculation. And then realized the calculation of severe dry–wet cycles. The calculation procedure is shown in Fig. 7.

## Results

### Unsaturation-saturation seepage results

**Figure 8:**
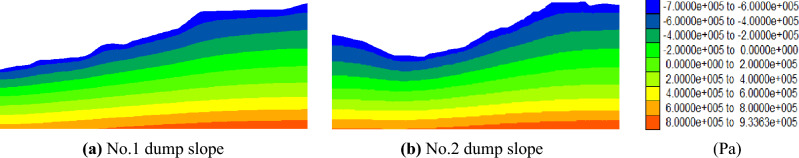
The distribution of pore water pressure for dump slope on the dry season.

The numerical model needs to be initialized in a non-saturated state during the seepage analysis before the dry–wet cycle. Figure 8 illustrates the pore water pressure distribution on the excavation slope during the dry season. The contour line of pore water pressure value 0 denotes the groundwater level, with saturated soil located below the groundwater level and having positive pore water pressure, while unsaturated soil is located above the groundwater level and has negative pore water pressure.

**Figure 9:**
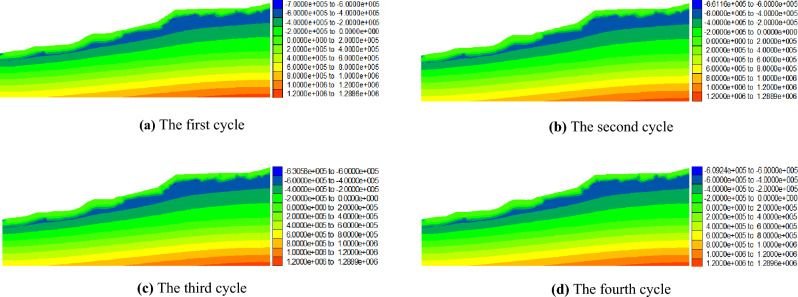
Pore water pressure distribution of No. 1 dump slope under dry–wet cycles (Pa).

**Figure 10:**
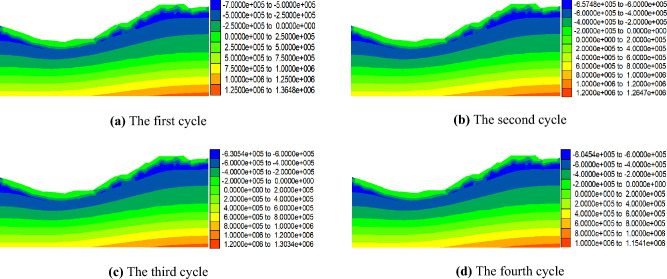
Pore water pressure distribution of No. 2 dump slope under dry–wet cycles (Pa).

Figures 9 and 10 present the distribution of pore water pressure in the No. 1 and No. 2 dump slopes, respectively, under dry–wet cycles. A comparison between Figs. 9, 10, and Fig. 8 shows that the surface soil of the dumping slope becomes saturated after four dry–wet cycles. The pore water pressure in the saturated zone of the dumping slope continuously increases until it reaches 0 kPa, while the pore water pressure in the unsaturated zone below the slope surface correspondingly increases from − 700 kPa.

**Figure 11:**
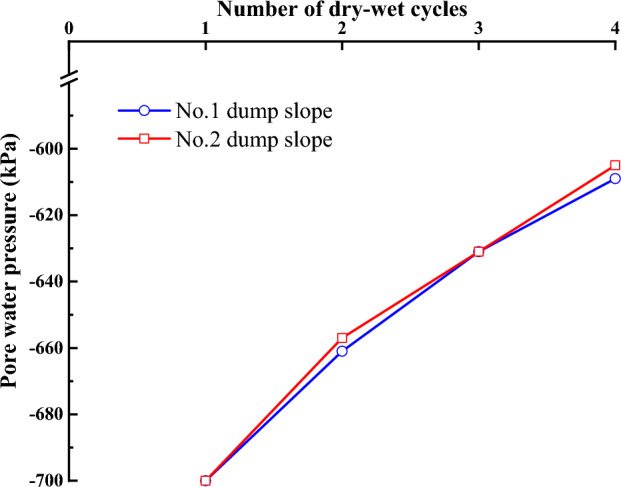
The variation curve of pore water pressure of dump slope.

Figure 11 illustrates the changes in pore water pressure with increasing dry–wet cycles for the No. 1 and No. 2 dump slopes. According to the figure, it is evident that the pore water pressure in the unsaturated zone of the No. 1 slope increased from − 700 kPa after the first cycle to − 661 kPa, − 631 kPa, and − 609 kPa after the 2nd, 3rd, and 4th cycles, respectively. The same trend can be observed for the unsaturated zone of the No. 2 slope, where the pore water pressure increased from − 700 kPa after the first cycle to − 657 kPa, − 631 kPa, and − 605 kPa after the 2nd, 3rd, and 4th cycles, respectively. These results indicate that the unsaturated zone below the slope surface in the dumping area gradually diminishes as the number of dry–wet cycles increases.

### Vertical displacement of the dump slope

**Figure 12:**
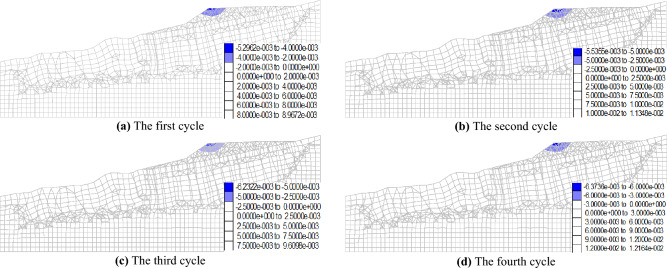
Maximum vertical displacement of No. 1 dump slope under dry–wet cycles (mm).

**Figure 13:**
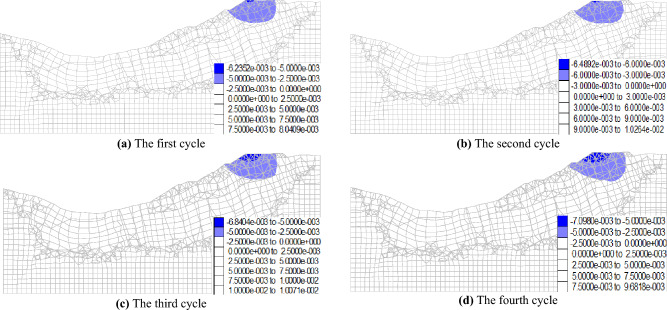
Maximum vertical displacement of No. 2 dump slope under dry–wet cycles (mm).

Figures 12 and 13 illustrate the maximum vertical displacement distribution of the No. 1 and No. 2 dump slopes under dry–wet cycles. As shown in the figures, the maximum vertical displacement location of both slopes is at the slope top after experiencing the dry–wet cycles, which can be attributed to the steeper slope angle that is more susceptible to deformation under dry–wet cycles. For the No. 1 slope, the vertical displacement data after each dry–wet cycle were 5.3 mm, 5.5 mm, 6.2 mm, and 6.4 mm. Similarly, for the No. 2 slope, the vertical displacement data after each dry–wet cycle were 6.2 mm, 6.5 mm, 6.8 mm, and 7.1 mm.

### FOS of the dump slope

The FOS of slope is calculated using the shear strength reduction coefficient method, utilizing Eq. (11). Through numerical calculations under varying cycle numbers, the FOS of the dump slope can be determined.

**Figure 14:**
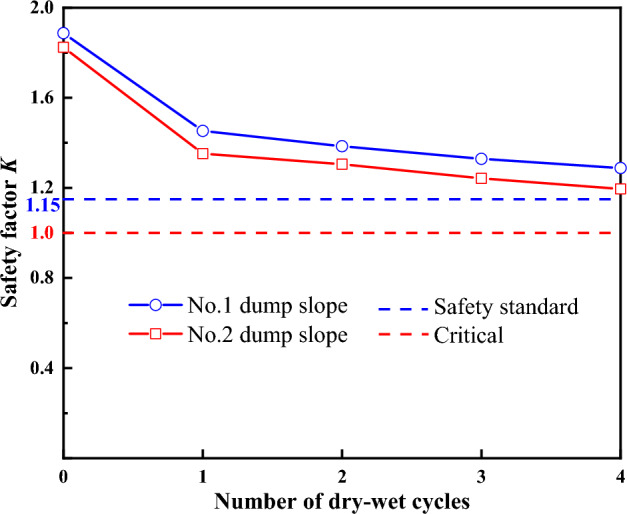
FOS *K* of dump slope under dry–wet cycles.

Figure 14 displays the variation curve of the FOS *K* of the dump slope after experiencing four cycles of wet and dry conditions. It can be observed from the graph that the FOS *K* values of the No. 1 and No. 2 dump slopes in the dry season are 1.887 and 1.824, respectively. Following four cycles of wet and dry conditions, the FOS *K* values of No. 1 dump slope are 1.453, 1.385, 1.318, and 1.281, respectively. Similarly, the FOS *K* values of the No. 2 dump slope are 1.352, 1.305, 1.242, and 1.195, respectively. The FOS of the slope curve shows a continuous decreasing trend with increasing dry–wet cycles. The FOS *K* value of the dump slope decreased the most after the first cycle of wet and dry conditions, and then the decreasing range of the FOS *K* value decreased, indicating a slow decreasing trend. This suggests that the cycle number's influence on the FOS is weakening. However, even after the fourth cycle of wet and dry conditions, the FOS *K* value still showed a decreasing trend and decreased to a minimum value.

### Comparative analysis of results

The actual vertical displacement value of the dump slope is monitored by (Differentia-Interferometric Synthetic Aperture Radar, D-InSAR). It is compared with the vertical displacement value of the dump slope obtained by numerical calculation. The accuracy of the numerical simulation results was verified.

**Figure 15:**
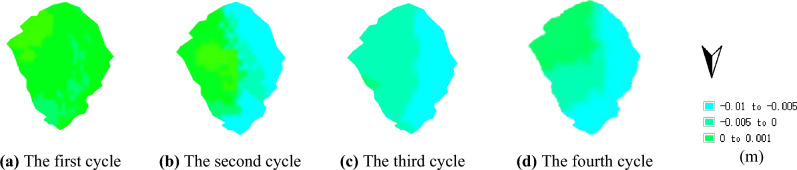
Vertical displacement of No. 1 dump slope.

**Figure 16:**
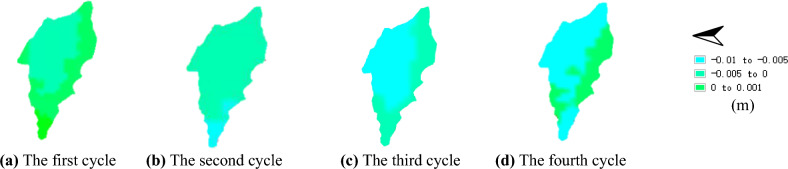
Vertical displacement of No. 2 dump slope.

The preceding section delineated the temporal extent over which the strength properties of dry–wet cycles were ascertained on four separate occasions. The chosen dataset for analysis involved Sentinel-1A imagery of the designated excavated slope that incorporated the temporal bounds of the strength above properties. Specific details pertaining to the image dataset are presented in Table 3. Through the execution of interferometric processing on the Sentinel-1A dataset of the dump slope, the vertical displacement distribution of dump slopes No. 1 and No. 2 was established. The resultant vertical displacement of dump slopes No. 1 and No. 2 are depicted in Figs. 15 and 16, respectively.

**Table 3:** Sentine-1A differential interference pair information.

Number of dry–wet cycles	Master image	Slave image	Time baseline (d)	Spatial baseline (m)	Incident angle (°)
1	2021/05/28	2021/06/09	12	− 13.656	42.110
2	2021/07/03	2021/07/15	12	− 6.516	42.123
3	2021/08/20	2021/09/01	12	− 42.573	42.120
4	2021/09/01	2021/09/13	12	− 44.365	42.113

The maximum vertical displacement values were collated and juxtaposed against the topmost vertical displacement value of the dump slope (Figs. 15, 16). Subsequently, a cumulative comparison curve of the maximum vertical displacement data was plotted while factoring in the influence of four dry–wet cycles.

**Figure 17:**
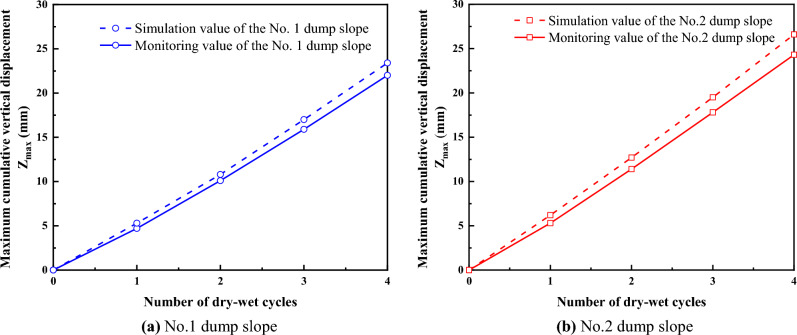
Maximum vertical displacement of dump slopes under the dry–wet cycles.

Figure 17 portrays the cumulative maximum vertical displacement data of dump slopes No. 1 and No. 2 under dry–wet cycles. The curve detailing the cumulative maximum vertical displacement data versus the number of dry–wet cycles highlights that the monitoring and simulation data of the dump slope showcase a persistent increase with the rise in the cycle number. Upon the conclusion of the fourth dry–wet cycle, the vertical displacement *Z*_max_ of the dump slope peaked and exhibited a continual upward trend. Furthermore, it was ascertained that the cumulative maximum vertical displacement data *Z*_max_ derived from both actual monitoring and numerical calculation demonstrated a striking level of conformity in their upward trends.

Table 4 illustrates the relative errors between the monitored data and simulated data of the No. 1 and No. 2 dump slopes. The numerical calculation outcomes are somewhat conservative and exhibit fewer relative errors when compared to the monitored data. This finding substantiates the dependability of the numerical simulation calculation method.

**Table 4:** Relative error of vertical displacement of dump slopes.

Number of dry–wet cycles	No. 1 dump slope			No. 2 dump slope		
	Simulated data (mm)	Monitoring data (mm)	Relative error (%)	Simulated data (mm)	Monitoring data (mm)	Relative error (%)
1	5.3	4.7	12.8	6.2	5.3	17.0
2	10.8	10.1	6.9	12.7	11.4	11.4
3	17	15.9	6.9	19.5	17.8	9.6
4	23.4	22	6.4	26.6	24.3	9.5

### Safety state evaluation

The FOS can accurately reflect the safety state of the dump slope. The FOS of the in-service dump slope is significantly affected by environmental conditions (such as dry–wet alternation and dry–wet cycles), and its value is dynamically changing, which can effectively reflect the safety state of the in-service dump slope. Therefore, using the FOS *K* as the evaluation criterion, the safety state of the in-service dump slopes of No. 1 and No. 2 were evaluated under severe dry–wet cycles, according to the high-altitude dump slope safety state evaluation standards. The complete safety stability standards for this safety state evaluation are presented in Table 5^18^, revealing that the safety stability standard value of 1.15 is the benchmark value for evaluating the dump slope under this safety state.

**Table 5:** Overall safety and stability standard of dump.

Working condition	Secondary dump slope safety stability standard	Southern dump slope safety stability standard
Natural condition	1.20–1.25	1.25
Rainfall condition	≥ 1.10	1.15

The FOS of the operational dump slope was matched against the safety stability standard for safety state evaluation. Under severe dry–wet cycles, the FOS of the No. 1 and No. 2 dump slopes surpassed 1.15, satisfying the safety stability standard. Table 6 shows the safety state evaluation outcomes of the dump slopes under dry–wet cycles, indicating that the in-service dump slopes of No. 1 and No. 2 remain in a stable state despite the harsh conditions.

**Table 6:** Evaluation results of safety state of dump slope under the dry–wet cycle.

Number of dry–wet cycles	FOS *K*			Stability	
	No. 1 dump slope	No. 2 dump slope	Safety and stability standard	No. 1 dump slope	No. 2 dump slope
1	1.453	1.352	> 1.15	Stable	Stable
2	1.385	1.305	> 1.15	Stable	Stable
3	1.329	1.242	> 1.15	Stable	Stable
4	1.288	1.195	> 1.15	Stable	Stable

### Summary of the results

Unlike slope stability problems caused by conventional rainfall at low elevations, this study considers the unique phenomenon of severe dry–wet cycles at high elevations. The effect of severe dry–wet cycles on the stability of the dump slopes was investigated. It was found that the pore water pressure and vertical displacement kept increasing with the number of dry–wet cycles, while the factor of safety kept decaying. The trends of the three curves all reflect the negative impacts of severe dry–wet cycling on the stability of the dump slopes from different perspectives.

## Conclusion

The unique severe dry–wet cycle phenomenon in high-altitude areas is included in the study. Focusing on the problem of safety and stability of high-altitude dumps, which are affected by the severe cycles of dry–wet, the main conclusions are as follows:

1. Due to dry–wet cycles, the dump slope surface soil transitions from an unsaturated state to a saturated state, leading to a continuous decrease in the unsaturated zone of the slope. Consequently, the pore water pressure gradually increases, and the matric suction below the wetting front redistributes according to the gradient.
2. The simulated vertical displacement data of the dump slopes under dry–wet cycles show minor errors and agree well with the actual monitoring data, confirming the reliability of numerical calculations for dry–wet cycles. With the increase in the times of dry–wet cycle, the No. 1 and No. 2 dump slopes experience vertical displacement, which continues to increase and develop towards the deeper layer.
3. While the FOS of the dump slopes demonstrates a nonlinear decreasing trend with the increase in the times of the dry–wet cycle, the FOS of both No. 1 and No. 2 dump slopes remain greater than 1.15, satisfying the safety stability standard.
4. The safety state evaluation results of the dump slope indicate that the in-service dump slopes of the cement-grade limestone mine exposed in the Ma Mu mining area, Sangri County, Shannan City, Tibet, China, remain in a stable state after four severe cycles of dry–wet.

## Data Availability

The data that support the findings of this study are available from the corresponding author upon reasonable request.
